# Book Reviews

**Published:** 1820-06

**Authors:** 


### CRITICAL ANALYSIS

OF

### RECENT PUBLICATIONS, IN THE DIFFERENT BRANCHES OF MEDICINE AND SURGERY;

### SELECT MEMOIRS, AND HISTORIES OF CASES;

### In the Literature of Foreign Nations.

---

Πατρίδες ἄρα
Ανδράσιν, οὐ πατραῖς ἄνδρες, ἀγαλλόμεθα.

---

*Medico-Chirurgical Annuary of the Civil Hospitals and Infirmaries of Paris; or, a Collection of Memoirs and Observations by the Physicians and Surgeons of those Establishments.* 4to. pp. 636; with fourteen Plates. Paris, 1819.

[In continuation from page 421.]

TWO cases by M. FOUQUIER, one of the physicians to La Charité, are the next in order in this work. The first of them is one which he terms

### *A Case of Febrile Nervous Dyspnœa, and Vesiculo-cartilaginous Degeneration of the Pulmonary Tissue.*

The history of this case is devoid of useful information, without an accurate account of the state of the lungs after death. M. Fouquier merely states, that the pleura was of the healthy appearance, "but the lungs were filled with vesiculo-cartilaginous grains of the size of a millet-seed, which did not prevent the intermediate tissue crepitating on pressure." This description cannot be satisfactory to any one who knows how to appreciate the importance of pathological anatomy.

The other case related by this physician, is one of *Nervous Paralysis.*

A girl, nineteen years of age, very large in stature and of a strong constitution of body, in whom "the sanguineous and lymphatic systems predominated," and whose menses appeared regularly, though only small in quantity, experienced a loss of strength in the lower extremities, three days after having menstruated in the ordinary manner. The upper extremities soon afterwards lost the power of voluntary motion. She perceived a beating noise in the ears, and suffered attacks of vertigo. The intellectual faculties were not at all deranged. Leeches were applied to the neck and anus. On the following day, she had entirely lost the power of moving the lower extremities: the arms still obeyed, to a certain point, the influence of the will. Sensibility remained in both of them. The patient complained of beating in the head and ringing in the ears. Her face was red and a little tumified, the pupils were dilated, and the lips were drawn to the left side and a little upwards. The tongue was covered with a greyish fur, and was turned a little towards the left side when it was protruded from the mouth. The pulse was full and strong, but not frequent. Abstraction of blood repeatedly, purgatives, and a vesicatory to the nape of the neck, were resorted to; but the patient died on the eighth day from the attack of the disease. The body was opened on the following day. "The vessels on the exterior of the brain, as well as those of the plexus choroides, were full of blood; and this fluid presented itself in a great number of points on the removal of slices of the brain. The texture of this organ was firm. There was no effusion in the ventricles, nor in the substance of it; neither was there any in the vertebral canal, which I had opened (says M. Fouquier) before my eyes."

We can offer no reasonings on this case at all calculated to clear up the difficulties it presents to us. The state of congestion of the blood-vessels of the brain may account for the paralysis, but then it is difficult to conceive that such a state of general congestion of the brain as would be productive of this, would not have caused disorder of the intellectual faculties. The state of the spinal marrow, which might be supposed to be the seat of the disease on which the paralysis depended, is indeed not described in a satisfactory manner. To endeavour to explain the phenomena in question on such a foundation, would be to seek in the clouds for what perhaps was under our feet. M. Fouquier seems to have very rude notions about pathological anatomy, or he could not suppose that his imperfect observations were likely to satisfy the generality of enquirers; or that we can, like him, complacently expatiate on mere words devoid of precise ideas, and say that there are cases of apoplexy and palsy which are "*purement nerveuses*;" and that this was one of them.

M. Chomel, a physician *attached* to La Charité, has contributed the history of "*a Case of remarkable Reunion* [*Union*] *of different Maladies in the same subject*," which we pass over, as presenting nothing remarkably interesting, or any grounds for useful inferences.

The next paper is by M. Kapeler, assistant-physician to the hospital Saint-Antoine, giving an account of some cases of *Painter's Colic*, treated in a way which he proposes as superior to that employed at La Charité, which has been celebrated for the cure of that disease ever since its foundation in 1602, as we find by the writings of Bordeu. We did not observe any cases in it during the time we visited it, but we believe it is still treated there by bleeding and antimonials. The means employed by M. Kapeler, are purgative medicines administered by the mouth, purgative and emollient glysters, fomentations of the abdomen, a grain of opium at night, and linseed infusion with broths for diet: measures that would be novel to no English nurse of ordinary intelligence and extent of observation.

M. Kapeler has also supplied an account of *a Case of Paralysis of the inferior Extremities without Vertebral Lesion, treated with success by the Production of two Issues in the Loins, after many other means had been employed without advantage;* and another, of *Paralysis of the Arms and lower Extremities without Vertebral Lesion, treated*

*with success by the application of a Seton in the Neck, and by two Caustic Issues in the Loins.*

The paralysis occurred in the first case, after the poor fellow who was the subject of it had undergone a long and desperate state of salivation; and, in the second, after a fall from a building on a grass-platform on the left shoulder and side. M. Kapeler very complacently states there was no *vice vertebral* present; but we are so rude as to doubt the truth of his assertion. We cannot spare sufficient space to give the history of the cases.

M. Beauchene, of the same Hospital, relates, in the first instance, a *Case of extraordinary Contraction of the Canal of the Urethra, cured by the use of Catgut and common Bougies.* This case would present nothing interesting to English surgeons, who are generally well acquainted with the use and efficacy of the catgut-bougie in cases where the canal is nearly obliterated by strictures.

M. Beauchene also presents a case of *Pott's Disease in a very advanced Stage, cured by the repeated application of the Caustic.*

The foregoing observations are devoid of novelty; and three others, which he relates, of great destruction of the soft parts of the face by cannon-balls, may be added to the list of horrible mutilations of this kind from which patients have recovered.

We now arrive at a paper by Alibert, comprising

*Some Considerations on Prurigo Formicans.*

Those of our readers who are acquainted with the talents of the author and the style of his writings, will readily suppose that he has drawn an interesting picture of the horrible malady that has here engaged his pen. Such is, indeed, the case; but we can, nevertheless, adduce from it but very few distinct observations of much importance, in regard to novelty, to English readers. Before we commence with the remarks of M. Alibert, we are induced to give a short account of a case that occurred to our observation, and which we had occasion to contemplate in a very particular manner, that, we think, tends more to elucidate the nature of this terrible malady than any one before recorded.

The subject of it, originally of the sanguineous temperament and of a healthy constitution, having never suffered any remarkable disease except small-pox from inoculation, measles, and hooping-cough, began, in the sixteenth year of his age, to devote himself to literary study with extraordinary ardour, and such assiduity, that, for the ensuing five years, he spent on an average, for the whole period, ten hours daily in acquiring languages, and mathematical and physical knowledge. At the end of this time, though pallid, languid, very thin in form, and almost devoid of appetite for food, he suffered no remarkable disease, until the approach of winter, when he became afflicted with prurigo formicans. It was not so severe now as it afterwards became, and only occurred about midnight, after more than ordinary sedentary and studious habits during the preceding day, and it disappeared on his becoming warm in bed. No eruption whatever took place on the skin, except from friction, when small red pimples were readily produced, which always subsided in a few hours, and never presented any thing like vesicular heads. A warm-bath always temporarily removed the paroxysm. On the contrary, on his going out one cold evening with only silk stockings on, the prurigo became so intense in the legs, that he arrived at the house he visited almost frantic from the suffering he had experienced; but it disappeared in a few minutes in a warm room. The malady continued in this way during the whole of the winter, and returned in the three successive ones, in a similar manner; during which period he had been pursuing the study of medicine in several parts of Europe, always devoting many hours daily to sedentary study, especially during the night when the day had been passed in more active pursuits.

On the approach of the fifth winter, when he was in London, the malady recurred with increased severity. It generally came on in paroxysms, about eight or nine o'clock in the evening, and continued during the greater part of the night, unless he went to bed, when it always disappeared in an hour or two. Had the whole body been covered with cowhage, his sufferings could not have been more severe. During the early part of the day, but little or no uneasiness was experienced, except any part of the skin usually covered by clothing were exposed nearly or quite to the cold air, when it always occurred in the part thus circumstanced. The skin had the healthy appearance, except when the pimples before described were produced by friction; and, from a firm determination to avoid this nearly or totally, no permanent eruption, nor any thing like excoriation, ever occurred. The warm-bath produced temporary relief, as well as washing the body with warm alcohol. Sudden active bodily exertion, so as to produce heat of the skin, would sometimes produce the prurigo; but it more constantly arose in this way from the impression of cold; and it seemed to be only where pimples were present, that the prurigo arose under the former circumstance. The health of the patient had now much declined. He became extremely languid and emaciated, and took but very little food, from want of appetite; though there was no other remarkable disorder in the functions of the alimentary canal. He passed daily about two gallons of limpid urine, similar to that often voided by women after a fit of hysteria, though his drink did not exceed three or four pints. The urine in its properties was like healthy urine diluted in a large quantity of water. He also became at this time partially deaf. The tonic barks produced but slight amelioration: no other medicines were resorted to. In the ensuing spring he altered for a time his usual habits, and spent a few months in the country, when the inordinate secretion of urine and the prurigo disappeared about the same time. Since then he has been obliged to use more active and regular bodily exercise, and has enjoyed somewhat more vigorous health; during which time, a period of five years, the prurigo has not returned, except occasionally, in a slight degree, after unusually intense mental exertions and almost total avoidance of sleep for many days in succession; and it has on these occasions been generally accompanied with the abundant evacuation of limpid urine, and with an increase of the dullness of the auditory faculty, from which he has never recovered.

We leave the above observations to the reflection of our readers; and shall now proceed to notice some of those of M. Alibert. He would, he says, in vain attempt to trace a picture of this desolating affliction: there are some evils which are above the power of language to describe, and this is one of them. Some patients become delirious from their sufferings; and one man ended them with a pistol, on his return from the waters of Cauterets, which had been equally inefficacious with all the means he had before resorted to. M. Alibert says, he has most frequently seen the affection in a continued form, but ordinarily having paroxysms of increase in the evening, and towards three o'clock in the morning. His description of the eruption which ordinarily attends it agrees with that of WILLAN. He considers the excoriation as dependant on the removal of the heads of the pimples by friction, but he does not state whether or not he believes the pimples themselves to be a consequence of the friction. This we believe to be the case generally; it was so certainly in that above described. The prurigo, the author states, often has intermissions of three or four hours' duration, especially when the patient eats, or is absorbed by some urgent occupation. Sometimes an attack continues only for five or six minutes, and then disappears for several days. He has seen some cases in which it occurred only in the soles of the feet. The most painful is that which attacks the genital organs of both sexes. Another form of it is that of old people, when it is often attended with ringing in the ears, weakness of sight, cramps, lassitude, and great derangement of the digestive functions: they often soon sink exhausted under their sufferings. We knew one patient of this kind who was much alleviated by sponging the body with alcohol several times a-day, after he had tried a multitude of things without deriving any advantage from them. He had before this insisted on being treated as if he had the itch, though he had for years been surrounded only by old domestics, and every way out of the reach of such an infection. Sulphur, externally and internally, afforded no relief. Lotions of decoction of white hellebore were of but little efficacy. M. Alibert has seen some cases where the muscles of the limbs have become so tense and contracted from the irritation, that the patient lost the power of moving them.

In one case mania, and in several others a state of fatuity, has been observed by him to alternate with the prurigo.

Its effects on the skin are not always the same. When it has not been very intense, and has affected the fine skin of women and children, it often disappears without leaving the least traces of its existence; but, when it has existed for a long time in a hard and rough skin, as that of old men, the epidermis often exfoliates like that of serpents, or acquires a hard and coriaceous consistence.

"The external causes of the prurigo formicans," says the author, "are very numerous. Forced labours, fatigues, watchings, &c. give immediate activity to the circulation, and may in time develop this terrible affection." In this explanation we do not agree with the author; from the case given at length above, which we had the means of observing in its origin and progress in the most intimate manner, and several others which we have examined with great care, from the particular interest that case excited about this malady, we think the prurigo is attended with diminished rather than increased activity of the circulation, especially in the skin; as is shown by its occurrence especially during the winter, by paroxysms being produced on exposing the body to cold air, or by great exhaustion; by its being often relieved by the warm-bath and stimulating applications; by its so commonly occurring in very old persons, &c.

We proceed with M. Alibert's further remarks on the causes of this affection. "A man, whose occupation was to draw floats of wood along a river, ceased to suffer from this malady after he had enjoyed a few days of repose at the Hospital Saint-Louis; but, as soon as he returned to his labour, it re-appeared. A courier of Paris experienced the same thing: he never suffered any uneasiness of this kind when he was at rest. The habitation of humid places not well supplied with fresh air, the abuse of spirituous liquors, the use of salt and corrupted meat, are causes of not less influence in its production. Almost all the subjects of this disease who come to the Hospital Saint-Louis, are idle and intemperate men, who pass their life in taverns, and whose general regimen is bad in almost every respect. It seems indeed that this desolating malady gives rise to a sort of passion for injurious articles of diet. One man, who had been a prey to it for five-and-twenty years, manifested a very particular depravation in his appetite: he sought with extreme avidity those aliments prepared with garlic, gherkins, vinegar, mustard, and other analogous substances. It may become developed under severe moral impressions. Theresa Delille lost her husband, and with him all the means of comfortable subsistence: she thence suffered deep mental affliction, very severe hemoptysis, which terminated, after six weeks, by appropriate treatment. She had a difficult convalescence; pains in her limbs, profuse sweats, with intermissions of two or three hours in their appearance, and suppression of the menses. The prurigo then appeared about the neck, back, and shoulders. Hardly any pimples could be seen on the skin. This woman could not resist scratching herself. The paroxysms of prurigo recurred several times a-day, after indeterminate intervals. She said that, when she resisted it without friction, it continued longer, and occurred with greater severity. It was efficaciously combatted by the use of mucilaginous baths."

M. Alibert advances nothing of importance respecting the treatment of this malady; though he states that he has seen some cases affecting vigorous subjects cured by the warm-bath, and one in a girl, occurring with amenorrhœa, by the same mean and blood-letting. It is commonly rebellious to all the measures that have been resorted to, when old persons are its subjects. He abrogates the idea of any topical application directed to the suppression of the local effect. His general practice is to give an emetic, purgatives, and make the patient submit to a light simple diet. "It can hardly be believed," he says,

"how hurtful all stimulants are in this malady." This statement is by no means universally true. The old man whose case is alluded to above only found relief in the constant use of wine inwardly, many times a-day, and in spirituous fomentations; and we have seen other cases occurring in persons exhausted by fatigue, especially by great mental exertions, who derived relief only from such measures. M. Alibert much favours the use of emollient baths. Lotions composed of hydro-sulphurous liquids, have been generally found injurious; alkaline and saponaceous baths, as those of the waters of Plombieres, are more salutary. In some cases, ointments composed of different preparations of the precipitates of mercury, have sometimes been of utility.

Professor Richerand has contributed a *Description of a new Process for the Extirpation of Cancers from the Lips,* which consists entirely in the use of curved scissars instead of the bistoury. The relative advantages or disadvantages of the scissars in regard to the bistoury, rest on the same grounds in this operation as in that for hare-lip; and, in spite of the prevalent dislike to the former instrument, we have seen enough of its application to make us prefer it. We are confident that, when the scissars are very sharp, the wound they make in such a part as the lip will heal as soon as one from the bistoury; and it may certainly be made by them with more determinate precision than by the latter instrument.

*Observations on Necroses of the Cranium, produced by Syphilis, or complicated with this Malady.* By M. Cullerier, Surgeon-in-Chief of the Hôpital des Veneriens.

M. Cullerier has here collected some interesting histories, but they do not present any thing new in regard to pathology. The principal facts they show in this respect are, that necrosis of the skull from the cause above designated may arise either from the periosteum becoming first diseased, or originate in the bone itself whilst the periosteum yet remains apparently healthy. They show, too, to what a great extent the cranium may be destroyed by caries, without the subjacent portion of the dura mater being obviously diseased; and they substantiate the fact, that, after the separation of large portions of the bone, the dura mater will become ossified. This occurred to a great extent in one case: a large space devoid of the natural bone, rising and falling with inspiration and expiration, became at length as firm and resistant in appearance as the surrounding bone; and, in another, spots of recent ossification were found dispersed in a portion of dura mater, denuded of the skull, after death. The reason why we do not find this happen after the use of the trephine for simple fractures of the skull, is, probably, that the dura mater is not kept in a state of irritation sufficiently long to cause it to ossify. It is a curious fact, that ligaments adjacent to bones, as well as the periosteum, become ossified, if a certain degree of irritation be long maintained in them. It is very common to find the dorsal part of the spine of horses who have long borne heavy burthens form one continuous piece of bone, with the ligaments about it also ossified. Instances of this are frequently found at the Veterinary College; several specimens of which we have examined.

The great object of M. Cullerier, in the paper under consideration, is to advocate the practice of removing carious portions of the skull by the trephine or the chisel and mallet, under certain circumstances; and he relates several cases in which he resorted to it, evidently with benefit and the most favourable results. The histories of these cases are necessarily long, and the utility of them would be lost in an abstract: we shall therefore pass them over at present, and select at a future time some of the most remarkable of them, which we will transcribe in detail.

The nephew of the above surgeon, and who is assistant-surgeon to the Hôpital des Veneriens, has contributed some

*Reflections and Observations on some Practical Points in Venereal Diseases.*

The cases alluded to only serve to prove the great utility and efficacy of caustic applications to primary venereal sores when they become stationary, or assume a sloughy or phagedenic character, after having proceeded to cicatrization to a certain extent under a course of mercury.

The two preceding papers do much credit to the talents of their authors; and we pass them over in this way, because we must confine our abstracts to what is novel as well as good.

Some cases follow by M. Nicod, surgeon-in-chief to the hospital Beaujon, which we shall get rid of as quickly as possible; for it is not agreeable to dwell on the writings of a man who cannot take up his pen without sneering at his contemporaries, and who is indebted to his office as surgeon to an hospital for any attention that may be given to any thing written by him. We shall have occasion to notice, in one of his cases, the miserable state of his reasoning faculties.

The first case he adduces an account of, is one of *Fracture of the Humerus, produced by Muscular Action.*

This occurred in a man, thirty-two years of age, of "a bilious temperament," a carpenter, whilst making a hole in a wall with an auger. The left hand was strongly pressed against the extremity of the handle of the instrument, whilst it was turned with the right. It was the left humerus which became fractured. He had suffered, for a month before the accident, severe pains in the arm, that he called rheumatic, but which did not prevent him pursuing his occupation. On the fortieth day after the accident, the consolidation of the uniting medium was found to be complete; and on the fiftieth the patient returned to his labour.

Another man fractured the humerus a little above the middle, on throwing a stone to some distance before him. He had never had the venereal disease, and had always enjoyed good health until about a month before the accident, when he began to suffer pains in the affected arm, that prevented him continuing his agricultural labour. Fifty days after the accident, a little fluctuation was felt about the fractured part, and three splinters of bone removed from it, by means of an incision made down them. The wound had healed at the end of about fourteen weeks from the time of the accident, and the patient returned to his labour.

The pains about the part in both these cases render probable the supposition, that the bone was in a morbid state previously to the accident.

Another case of fracture of this kind occurred in the clavicle. The patient was a woman, sixty-eight years of age. The accident happened from her suddenly putting her hand behind her, as she sat in a chair, the fore-arm being in a state of extreme pronation, to shut a drawer. She immediately felt pain about the clavicle, and it was soon afterwards found to be broken. On the thirtieth day from the accident, the union of the bone seemed to be solid, "although the callus was still much swelled;" and the patient left the hospital on the fortieth, being able to use the corresponding arm very well. Three months afterwards she returned, saying that she had not given her arm rest, and that the part over the fracture was red and tumid. An abscess occurred on this part: it was opened; the clavicle was carious and exfoliated to the extent of eight or ten lines. The wound at length cicatrized. "The callus appeared to become compact again, without any other medicines than bitter tisans and antiscorbutic wine." The patient again left the hospital, apparently cured. Some months afterwards she returned, with ulcers over different parts of the clavicle, on the shoulder of the same side, on the back, and on the anterior part of the chest. "Several periostoses on the ribs, (says M. Nicod,) and the aspect of the ulcers, did not permit me to doubt that the venereal virus was the remote cause of the fracture and the symptoms I witnessed. I sent the patient to M. Cullerier, surgeon-in-chief of the Hôpital des Veneriens, with a summary of my observations. The decrepitude of the patient announced the approach of death." There are some propositions, of which the folly is too gross to bear remark, and this is one of them. But we must inform our readers, that the above are all the reasons on which this surgeon founded his opinion that the disease of this old woman dying of decrepitude was syphilitic, and for which he thought proper to send the poor creature to end her days in a pox-hospital.

M. Nicod also relates a *Case of Fragility of the Femur and Humerus in a Woman affected with Cancer in the Breast.*

A woman, fifty-six years of age, who had a tumor in her right breast, that had ulcerated and cicatrized several times,* was brought to the hospital with the neck of the right femur fractured, occasioned by a fall from her bed fifty days previously. The bone had been reduced

* M. Nicod talks with a good deal of self-approbation of his having been the man who first discovered that cancerous ulcers would spontaneously cicatrize to a certain extent, in some instances. We wonder he has not claimed the merit of the discovery of the union of wounds by the first intention.

and treated in a proper manner, but no firm union had taken place. The limb was much shortened, and the thigh considerably bowed. Petit's distensive apparatus was applied. One hundred and ten days afterwards, the limb was found to be in nearly the same state as on the fiftieth. The patient had severe pains in the lower extremities, and still more severe in the upper, which she could not move. Three or four days after, she broke the humerus of the right side, at its middle, on turning in bed; and, about eight days subsequently, the left femur, just below the great trochanter, in the same way as the right one; but the patient declared that her limb was already bowed before she fell out of bed. She died in a little less than three months after her entry into the hospital, according to M. Nicod, "of a sort of typhous (adynamique) fever, arising from the commencement of a cancerous cachexy."

On dissection, "the left femur was found to have been fractured below the great trochanter; the ends of the bone were surrounded by a little blood, which appeared to have issued from the broken extremities; these did not present the development of the vascular tissue which is the prelude to the formation of callus, although the bone had been broken fifteen days: the femur was not softened, except a little at the surface of the fracture. Neither the left os ilium nor its cotyloid cavity, presented any remarkable alteration in their appearance. The right femur, from its neck to its middle, formed nothing but a hard, fleshy, lardaceous mass, interspersed with very thin osseous laminæ. This part of the femur was twice the natural volume; the trochanters were recognizable only by the more considerable mass they formed. The neck, where the first fracture seemed to have existed, was fibrous for the space of about half an inch, without presenting the smallest particle of bone. The head of the femur was not increased in size; its cartilage was in the healthy state; but the spongy part was so much softened, that it gave way to the slightest pressure. The cartilage of the cotyloid cavity was a little altered, especially in its border, which was tumefied, as well as the synovial gland. The triangular ligament was of a less firm consistence than in the healthy state. The crista of the ilium was a little softer than ordinary, but the rest of this bone was not at all altered in its structure. The middle part of the femur was healthy for the space of about an inch; but, from this part to the condyles, the bone was of twice the natural size, and was very easily penetrated by the scalpel. The condyles had not increased in bulk; their cartilages were healthy, but their spongy structure was softened.

"The fragments of the broken humerus were not united, and presented no callus: they were covered by fleshy granulations on almost the whole of their surface; some points only of their edges appeared to be in a state of necrosis, and as if they would have exfoliated had the patient lived longer. There was a little effusion of blood about the part, which seemed to have resulted from the friction of one part on the other in the motions of the arm. The medullary canal was filled up by a reticular tissue to the extent of about an inch from the fracture; the compact tissue of the bone was softened to the same extent, so that the scalpel penetrated it as easily as the spongy tissue above described. All the rest of this bone appeared to have preserved the natural hardness.

"The tumor in the breast presented nothing remarkable, more than a lardaceous mass, of a homogenous appearance until it approached the part which had been the seat of the ulcer."

We have thought it right to transcribe the above account in detail; for, though no important inferences may now be drawn from it, it may serve, with other analogous histories, to furnish the means for doing this at some future time.

The same surgeon then relates *a Case of successful Amputation of the Left Leg during Pregnancy, and of the perfect Cure of a Rupture of the Right Achillean Tendon in the same Subject.* The patient was in the eighth month of her pregnancy. She was brought to the hospital with a large and deep wound at the lower and internal part of the left leg: the extremity of the tibia was broken into a great many pieces; the cavity of the ankle-joint was opened, and the astragalus injured. The right leg presented, at the lower part of its posterior surface, a wound three inches in diameter, with a rupture of the Achillean tendon, and of the posterior tibial artery. These accidents arose from the bursting of a bomb in the room where she was sitting. The left leg was amputated; and the usual bandage for such an accident applied to the right.

No symptoms of a serious character ensued. The patient was delivered of a healthy boy, on the thirty-eighth day from the accident, after a labour of two hours' duration. The stump had completely cicatrized on the fifty-fifth day; and the wound over the ruptured tendon on the sixty-eighth. On the eighty-first she was obliged to leave the hospital, (which was taken possession of by the English army, in the autumn of 1815,) when she had two small wounds on the internal part of the foot not yet healed. She, however, began to walk with a stick; and on the ninetieth returned to her domestic occupations, the wounds having cicatrized, and the foot possessing the natural power of motion.

---

### *An Essay towards a Pathology of the Organ of Hearing.*

By Professor Rosenthal, Berlin.

In the present state of our physiology and pathology of the organ of hearing, we must seize with avidity every addition, however little, to our knowledge of these subjects; and hence this essay of Professor Rosenthal becomes very interesting: for, though it presents but little more than hints, these hints are such as may disclose some views of considerable importance to other observers.

Even the mechanical part of acoustics, which is the most perfected, leaves much to be desired, since it hardly teaches us to appreciate and measure the quantity of sound. If it teaches us that sound depends on the vibration of bodies; that these vibrations spring from the centre of the sonorous body, radiating uniformly on all sides, and converging or diverging according to the difference of the medium through which they pass, which gives rise, respectively, to low or loud sounds; it is, on the other hand, impossible for us to calculate exactly the refraction which these sonorous rays submit to; and we have less reason to be satisfied with the explanations that have been given of the nature of sound, its different degrees of intensity, and of articulated tones. As the knowledge of the causes which give rise to those particular modifications of sound, is of great importance in the explanation of the phenomena of audition, the author first examines how far the present state of acoustics really elucidates those circumstances. All that they teach us, with certainty, he says, in respect to these phenomena, is, that we cannot account for them, either by the succession of the vibrations whence arises simple sound, or by the extent of the same vibrations, or by the distance of the sonorous body; but that they consist in a modification of sound entirely different and distinct from all these particularities. The functions of different parts of the ear are yet but very imperfectly understood; and comparative anatomy, whence so much elucidation might be expected, has not done much in the way of explanation of them.

All the derangements of the sense of hearing, the author considers, may be referred to the three following principal forms:

1°. *Surdity*, (Lat. *surditas*, Gr. *cophosis*,) in which the faculty of hearing articulated sounds is entirely abolished.

2°. *Difficulty of hearing*, (*dysœcia*,) in which this faculty is so far diminished, that articulated sounds can be heard only by means of a peculiar apparatus.

3°. *Alteration or diminution of hearing*, (*paracusis*,) in which the faculty of hearing articulated sounds by the natural means is deranged by want of precision.

Surdity may be distinguished into two degrees: the first is marked by the absolute impossibility of hearing; (this degree is ordinarily connate, and leads to dumbness;) and the second, by the faculty of perceiving certain sounds, for examples, that of a whistle, the vowels,* &c.

The distinction of these two degrees is of great importance in practice, and especially in the treatment of the deaf and dumb, because we are very apt to fall into error, and mistake for the true auditory faculty the excessively delicate sense of touch which these individuals often possess. Some experiments of Pfingsten,† made with great care, prove this; two of which are here adduced in illustration of this remark.

A deaf-and-dumb girl, occupied in sewing in a room near the street-door of the house, always gave notice when any one opened or shut this door. A bell was attached to it, that, on every motion, made sufficient noise to be heard distinctly in the next room; but, as, with the exception of this noise, no shock or agitation could be per-

* F. Hoffmann relates an instance of a young man who could discern no other sound than that from winding a goat's horn. See Kritter and Lartin, *Ueber das schwere Gehoer*.

† See Pfingsten, *Vieljaehrige Erfahruug ueber die Gehoerfehler der Saubstummen.* Kiel, 1802; p. 32.

ceived, the phenomenon appeared to Pfingsten somewhat surprising. Wishing to know what the girl really experienced, he rang the bell loudly without opening the door: she did not seem to perceive any thing. He then had the bell made silent, whilst a person very cautiously opened and shut the door, which was done so gently that he himself did not perceive it; but the little girl immediately gave notice that some one had come in. On reflecting on these circumstances, he was led to consider, that the chair on which she sat communicated to her body a certain agitation which made her conscious of the motions of the door.

Another deaf person possessed the sense of touch in a still more delicate degree. This, a little girl, lied in the same chamber with the servant of the house, with whom she had, every evening, long conversations about her toilette or other subjects, after the lights were extinguished. She laid on her side, and placed her hands on the chest of the servant, who lay on her back, and thus was enabled to discern the discourse of the latter. Pfingsten, desirous to see himself this singular mode of intercourse, persuaded the two girls to engage in conversation in his presence; and he perceived that, when the deaf girl had placed her hands on the breast of the other, she could repeat correctly almost all the words the latter had pronounced.

These cases, and several others related by Pfingsten, also show that, when we have to ascertain the degree of surdity, we should take care to avoid the intervention of bodies strongly conductive of sound; and that, consequently, experiments with the above-mentioned view should not be made in a house, but always in the open air, and on a but-slightly conductive surface.

The author presents some useful observations on the appearances found on dissection in other cases; and he shows that they have not always been devoid of results precisely indicative of this malady. He says, it is proved that the affection carried to the degree of perfect deafness, depends more frequently on morbid states of the soft parts than on deviations from the proper structure of the solid structure of the ear. Thus, Frederic Hoffmann has found the auditory nerve in a state of atrophy in a deaf-and-dumb person, whose ear was in other respects perfectly well-formed. Arnemann has found it harder than ordinary; Haighton has seen it replaced, in the vestibulum, by a caseiform matter; Duverney and Sandifort have observed a steatomatous tumor, which strongly compressed it; and the observations of Itard have been conformable to those just designated. In one case, he found all the parts of the organ so healthy in appearance that he could attribute the deafness to nothing but paralysis of the nerve: in another, the affection depended on obstruction of the auditory passages; in a third, the cavity of the tympanum and the vestibulum contained some small calcareous concretions. He has also seen the former cavity contain a thick yellowish lymphatic fluid, or serum, enveloped in several cells formed by a membranous structure. Prof. Rosenthal has witnessed a case analogous to the latter; in which the auditory nerve was of so solid a texture, that it surpassed even the facial nerve in hardness. The whole of the medulla oblongata was also of a more firm consistence than ordinary. The Eustachian tubes were well formed, and contained, in the canals, which were open as far as the tympanum, a limpid yellowish fluid. The meatus auditorius presented nothing extraordinary with respect to its diameter, its length, and its direction; but the membrane of the tympanum was thicker than common. The vaulted part of the tympanum was no thicker than paper; and, immediately above the union of the incus with the malleus, the bony substance had been quite absorbed, so that the bone, in this point, appeared softened, and as it were membranous. Having removed this thin plate, which was of considerable extent on the left side, he found that the largest of the mastoidean cells, those which were in contact with the parietes of the tympanum, and the cavity of the latter also, contained a serous fluid, of the colour and consistence of that found in the Eustachian tubes. The periosteum was thickened in this part, from inflammation; and it formed several little areolas, which enveloped the small bones of the ear. The latter were constructed in the ordinary manner. The labyrinth presented nothing peculiar. The semicircular canals were constructed in the regular manner; and nothing extraordinary could be perceived in the manner in which the auditory nerve was distributed in the concha and vestibulum.

Instances have also occurred where the malady depended on a deviation from the proper structure of the solid parts of the organ: these cases however have been of but rare occurrence. Mundini has found the concha composed of only one convolution and a half. Dr. Baillie has seen the small bones in the tympanum three times less in size than ordinary. Valsalva has found the stapes firmly united to the fenetrum ovale; and Reimarus, a total absence of those bones.

The affections of the second class, dysœcia, present different degrees of difficulty of hearing. In the first degree, the patient does not hear a distant sound, and especially acute tones; but he discerns, indistinctly it is true, articulated sounds when forcibly uttered. In the second degree, he hears and discerns very well both acute and grave tones, and can discern words, but only when the voice is elevated.

The conditions of those two degrees of the malady are little known, because it may be admitted, with some certainty, that the proximate cause is some alteration of the auditory passage, or of the sensibility of the nerve, all the internal parts of the organ being in other respects well formed.

Amongst the alterations of the organs which conduct the sound may be ranged,

1°. The total obliteration of the external auditory canal by foreign bodies, its imperfect formation, or the entire absence of it. A superficial examination is, almost always, all that is necessary for the discovery of these cases; as the patient hears only when he holds solid bodies between his teeth, and his hearing is not diminished by the complete closure of the external meatus auditorius.

2°. Diseases of the tympanum, as inflammation of the membrane lining it; caries of its parietes; effusions of blood, pus, or serum, into its cavity. Inflammation and suppuration in this part are of more frequent occurrence than has been imagined, because the affection has been very often supposed to be rheumatism. "I have frequently found," says the author, "in the dead bodies of old persons, the membrane of the tympanum opake and thickened, without being able to account for this occurrence in any other way than by making it depend on previous inflammation."

When this degree of the malady depends on an altered state of nervous irritability, it becomes especially necessary, in the treatment of it, to have regard to the species of morbid excitement which has been present. The diagnosis must here depend on that of nervous affections in general; but the existence of this form of the affection may be inferred with tolerable certainty, 1°, when the patient had formerly been very sensible to the impression of certain tones or of sounds in general; 2°, when the faculty of hearing has suddenly disappeared, without any signs of inflammation; and, 3°, when it coincides with other nervous maladies.

The third class, that of cases of alteration or diminution of hearing, comprises various degrees of that faculty, descending from a state of hearing extremely perfect, which may be either connate or acquired by force of exercise.

As it is principally the cavity of the tympanum, and the parts it contains, which have most influence on the intensity of sound, and consequently perform an important part in the propagation of articulated sounds, it is those that we must here take into consideration, in order to understand the nature of this class of affections. Amongst the great number of alterations in the state of them, which pathological anatomy has disclosed, may be ranged the following:

1°. Alteration of the membrane of the tympanum; which may consist, either in a congenital malformation or improper situation of it, or in thickening, ossification, perforation, or rupture, of it: but there are no certain means by which those accidents may be discovered to exist in all cases.

2°. Repletion of the tympanum with some liquid. This is occasioned, more frequently than has been supposed, by obstruction of the Eustachian tube, by which the natural secretion is confined and collected in undue quantity. In this form of the malady, the patient hears the sounds of words uttered before him tolerably well, but a continual humming in his ears prevents him from discerning distinctly the articulated tones. This may be considered as a diagnostic character of it.

3°. Alterations of the membrane of the fenetrum ovale; amongst which may be mentioned faults in respect to its form and situation, as well as in the thickness of it.

But, as a difference in the intensity of the sound may also produce modifications in the sensations, the purely conductive organs should not here be lost sight of, such as the external ear and auditory canal; since it is those which regulate the mass of sonorous oscillations with which the auditory nerve is struck; although any aberrations from the natural state of them have but little influence on this sort of derangement of the sense of hearing. Thus, for example, WEPFER

relates a case in which the external ear was totally destroyed by an ulcer, and yet the patient did not hear less perfectly in consequence of it. Morbid conformation of the meatus auditorius, or alterations of the ceruminous secretion of this canal, appear to have more influence on the state of the function.

Lastly, the state of nervous action demands here especial consideration. Too little or too great a degree of it may equally render the perception of sound obscure. If it be not possible to distinguish those differences in particular cases, we may always obtain some knowledge tending to elucidate them, by paying attention to the state of sensibility in general in the individual.

---

